# Multiplex Real-Time PCR for the Detection of Tetracycline, Ciprofloxacin, and Erythromycin Resistance Determinants from Human and Foodborne *Campylobacter jejuni* and *Campylobacter coli*

**DOI:** 10.3390/microorganisms11122927

**Published:** 2023-12-06

**Authors:** Véronique Zeller-Péronnet, Nancy Bretschneider, Johanna Lausch, Nadera Hanifi, Melanie Pavlovic, Michael Zarske, Huong Quynh Luu, Ulrich Busch, Kerstin Stingl, Ingrid Huber

**Affiliations:** 1 Department for Food and Food Hygiene, Bavarian Health and Food Safety Authority (LGL), 85764 Oberschleissheim, Germany; veronique.zeller-peronnet@lgl.bayern.de (V.Z.-P.); nancy.bretschneider@lgl.bayern.de (N.B.); nadera.hanifi@lgl.bayern.de (N.H.); melanie.pavlovic@lgl.bayern.de (M.P.); ulrich.busch@lgl.bayern.de (U.B.); 2 National Reference Laboratory for Campylobacter, Department of Biological Safety, German Federal Institute for Risk Assessment (BfR), 10589 Berlin, Germany; michael.zarske@bfr.bund.de (M.Z.); kerstin.stingl@bfr.bund.de (K.S.); 3 National Institute of Veterinary Research (NIVR), Hanoi 100000, Vietnam; lqhuongvet@yahoo.com

**Keywords:** food safety, *Campylobacter* spp., food and clinical isolates, antimicrobial resistance determinants, susceptibility testing, real-time PCR assay

## Abstract

*Campylobacter jejuni* and *Campylobacter coli* are the predominant thermophilic species responsible for foodborne gastroenteritis worldwide. Elevated resistance to certain antibiotics was observed due to antimicrobial therapy in farm animals and humans, while reduced antimicrobial usage partially reduced antibiotic resistance. Monitoring the antimicrobial resistance demonstrated a substantial fraction of multi-resistant isolates, indicating the necessity of reliable tools for their detection. In this study, resistance determinants in 129 German and 21 Vietnamese isolates were selected to establish a novel multiplex real-time PCR (qPCR), facilitating the simultaneous detection of four resistance determinants. These comprised *tet*(O) gene variants associated with tetracycline resistance, point mutations GyrA_T86I and GyrA_T86V associated with ciprofloxacin resistance, and the *erm*(B) gene together with the point mutation A2075G in the 23S rRNA gene, associated with erythromycin resistance. Moreover, the performance of the qPCR assay was evaluated by comparing the results of qPCR to phenotypic antimicrobial resistance profiles, obtained with standardized EUCAMP3 microdilution panel, which showed 100% similarity (inclusivity and exclusivity). Variation in measurement methods, including qPCR machines and master mixes showed robustness, essential for laboratories. The assay can be used for the rapid detection of resistance determinants, and is beneficial for monitoring the spread of antibiotic resistance in *C. jejuni* and *C. coli.*

## 1. Introduction

*Campylobacter* is the most frequently reported foodborne bacterial pathogen in humans in the European Union [1]. Consumption of poultry meat contaminated with thermotolerant *Campylobacter* species can cause severe gastroenteritis. *C. jejuni*, followed by *C. coli*, are the predominant thermotolerant *Campylobacter* species in poultry samples and are mainly responsible for foodborne human infections [2,3]. The use of antibiotics in animal farming and for the treatment of human diseases promotes antimicrobial resistance. The relationship between antibiotic use and increasing occurrence of resistance has been frequently described [4]. In January 2022, a new Veterinary Medicinal Products Regulation (2019/06) was implemented throughout the European Union (EU) [5], which updated the rules on the authorization and use of veterinary medicines in the EU to preserve the effectiveness of antibiotics for the future. However, due to enhanced and prolonged antimicrobial usage in high selection areas, such as Southeast Asian countries, the prevalence of antibiotic-resistant *Campylobacter* on poultry meat and the risk of multi-drug resistance islands (MDRI) are increasing [6]. It is becoming crucial to identify resistance determinants independently of time- and labor-consuming phenotypic characterization and to develop fast tools for the use in European monitoring surveys of circulating resistance determinants.

Several studies addressing the impact of antibiotic usage on the formation of resistance have already revealed multiple resistance mechanisms, such as duplicated genes, mosaic genes, gene mutations, plasmids carrying resistance determinants, and transposons, all of which contribute to the spread of antibiotic resistance [4,7,8]. For ciprofloxacin resistance, the point mutation T86I in the gyrase A subunit is the most frequent resistance determinant in *Campylobacter* spp. [9,10,11]. Erythromycin resistance in *Campylobacter* spp. was shown to be mainly conferred by the point mutation *23S rRNA* A2075G [12,13]. However, in Asian countries [14], also sporadically in Europe (Spain) [15], and in the United States [16], the *erm*(B) gene, encoding a methyltransferase presents a second, highly transferable resistance determinant in *C. coli*. Tetracycline resistance in *Campylobacter* spp. is based on the presence of a ribosomal protection protein encoded by *tet*(O) [17] and mosaic variant genes [18,19].

Zarske et al. [20] investigated resistance determinants in German and Vietnamese thermotolerant *Campylobacter* spp. populations. Moreover, they demonstrated the presence of different resistance determinants, such as resistance genes, gene variants, and point mutations in distinct genes (*gyrA, 23S rRNA, rpsL*). Based on this genomic knowledge, worldwide prevalent resistance determinants were selected to develop a multiplex real-time PCR assay capable of covering resistance to tetracycline, ciprofloxacin, and erythromycin antibiotics.

In the last decade, polymerase chain reaction (PCR)-based detection systems have increasingly been applied to explore determinants of antimicrobial resistance among thermophilic *Campylobacter* spp. isolates. With combination of singleplex PCRs, the presence of two tetracycline resistance genes *tet*(O) and *tet*(A) can further be screened [21]. Laprade et al. [22] developed four conventional multiplex PCR assays that detect tetracycline resistance gene *tet*(O) in combination with virulence and toxin genes. A real-time PCR assay based on the amplification of a fragment of the 23S rRNA gene, surrounding bases 2074 and 2075, was developed to detect macrolide-associated mutations [23]. Additionally, Zhang et al. [13] identified the presence of the mutation in the 23S rRNA gene by mismatch amplification mutation assay (MAMA) PCR and DNA sequencing; for the presence of the *erm*(B) gene, a conventional PCR was applied. Zirnstein et al. [24] published a MAMA PCR assay, and Espinoza et al. [8] published a real-time PCR for the detection of the point mutation T86I in the gyrase A that is associated with resistance to ciprofloxacin.

In a further study, Nguyen et al. [25] characterized Vietnamese *Campylobacter* isolates in antibiotic susceptibility testing EUCAMP2 and identified resistance determinants, using MAMA PCRs for point mutations at positions 2074 and 2075 of the 23S rRNA gene, as well as for the screening of the point mutation T86I in the gyrase A. A specific conventional PCR was applied to detect the presence of the *tet*(O) gene.

In the current study, we developed a multiplex real-time PCR assay to simultaneously detect the presence of four resistance determinants in *C. jejuni* and *C. coli*. In these assays, the widely distributed resistance gene *tet*(O), *encoding the Tet(O) ribosomal protection protein* [26] and the point mutations T86I and T86V within the gyrase subunit A [8,24], were retained to screen tetracycline and ciprofloxacin resistance, respectively. In order to cover erythromycin resistance, two detection systems, including the resistance gene *erm*(B), encoding the Erm(B) ribosomal methyltransferase [13,27] as well as the point mutation A2075G in the *23S* ribosomal RNA gene [13,28], were selected. The selection of these targets for multiplexing the real-time PCR assay was based on the European Union Summary Report on Antimicrobial Resistance of EFSA and ECDC (2023) [29], which indicated that combined resistance to both ciprofloxacin and erythromycin is considered critically important for the treatment of campylobacteriosis.

A test panel consisting of 129 German isolates obtained from food and human sources, as well as 21 Vietnamese isolates derived from chicken feces and exhibiting thermotolerant characteristics were phenotypically tested for resistance to six antibiotic classes and used for the validation of the novel multiplex real-time PCR.

## 2. Materials and Methods

### 2.1. Campylobacter Isolates and Growth Conditions

A total of 68 human isolates of *Campylobacter (C.)* spp. were obtained from systematical screenings performed during the 2018–2023 period in stool samples from gastroenteritis patients at LGL, department of human bacteriology, as well as private laboratories in the south of Germany. A total of 61 food isolates of *Campylobacter* spp. were isolated at LGL or provided by the German Federal Institute for Risk Assessment (BfR) during the 2019–2022 period, mostly from chicken neck skins from slaughterhouses and chicken breast from retail shops. Vietnamese isolates were previously isolated from chicken feces [20]. The classical microbiological method to detect *Campylobacter* spp. was carried out according to ISO 10272-2:2017 [30]. Briefly, 1 mL meat rinse was spread onto the surface of three selective mCCD agar (modified Charcoal-Cefoperazone-Deoxycholate Agar, Merck, Darmstadt, Germany) plates and incubated at 42 °C for 44 ± 4 h with a concentration of 10% carbon dioxide (CO_2_). Subsequently, all isolates were identified at the species level by matrix-assisted laser desorption ionization-time of flight mass spectrometry (MALDI-TOF MS) using the MALDI Biotyper (MBT) platform (Bruker Daltonics, Bremen, Germany) according to Huber et al. [31] to ensure the identification of each isolate.

In total, 85 *C. jejuni* and 44 *C. coli* isolates from Germany were collected for this study. *C. jejuni* strain DSM 4688 (DSMZ-German Collection of Microorganisms and Cell Cultures GmbH, Braunschweig, Germany) and *C. coli* strain 2012-70-443-2 (Technical University of Denmark, Lyngby, Denmark) were used as a negative control strains for phenotypic resistance testing and the multiplex real-time PCR-assay. All *Campylobacter* isolates and strains were stored at −80 °C using the MAST Cryobank system (Mast Diagnostica GmbH, Reinfeld, Germany) and are listed in Supplementary Material Table S1.

These 129 *Campylobacter* isolates, together with 21 Vietnamese isolates, were characterized phenotypically and genotypically, and formed a test panel for the design, development, and validation of a real-time PCR assay. All isolates were phenotypically tested for resistance to six antibiotics in standardized microtiter plate format EUCAMP3. For genotypical characterization, an NGS-based approach was applied to identify different genetic determinants conferring antimicrobial resistance.

### 2.2. Antibiotic Susceptibility Testing EUCAMP3

The European standardized Sensititre™ EU Surveillance *Campylobacter* EUCAMP3 plate system (Thermo Fisher Scientific Inc., Waltham, MA, USA) was used to identify phenotypic resistance patterns of isolates from Germany and Vietnam against six antimicrobial agents: chloramphenicol, erythromycin, gentamicin, ciprofloxacin, tetracycline, and ertapenem. According to the European Union Summary Report on Antimicrobial Resistance of EFSA and ECDC [29], these antimicrobials have been reported to be mandatory for *C. jejuni* and *C. coli* as representatives of six different antibiotic classes of phenicols, macrolides, aminoglycosides, (fluoro-)quinolones, tetracyclines, and carbapenem, respectively.

Isolates stored at −80 °C were grown on Columbia agar (ColbA), supplemented with 5% sheep blood (Oxoid, Thermo Fisher Scientific Inc.) for 24 h with a concentration of 10% CO_2_ at 42 °C and subcultured once for additional 20 ± 2 h before antibiotic susceptibility testing. Isolates were inoculated at a bacterial concentration between 2 × 10^5^ and 8 × 10^5^ CFU/mL in cation-supplemented Mueller–Hinton broth (Thermo Fisher Scientific Inc.) with 5% fetal bovine serum (PAN-Biotech, Aidenbach, Germany) (CAMHB/FBS). A volume of 100 µL inoculated CAMHB/FBS (5 × 10^5^ CFU/mL) was added to each well of EUCAMP3 format plates, and the plates were incubated at 37 °C for 44 ± 4 h with a concentration of 10% carbon dioxide (CO_2_).

Minimal inhibitory concentrations (MICs; in mg/L) were determined using the semi-automatically Sensititre™ Vizion™ system (Thermo Fisher Scientific Inc.) and the Sensivizion V2.0 software (MCS Diagnostics BV, Swalmen, The Netherlands). Epidemiological cut-off values (ECOFFs, Table 1) for resistance determination were based on the European Committee on Antimicrobial Susceptibility Testing [32,33,34].

### 2.3. DNA Extraction and Quantification

*Campylobacter* isolates were subcultured on ColbA or Tryptone Soy Agar with Sheep Blood (Thermo Fisher Scientific Inc., Waltham, MA, USA) (TSASB) for 20 ± 2 h with a concentration of 10% CO_2_ at 42 °C. Bacteria were resuspended from agar plates in 200 µL phosphate-buffered saline buffer with pH of 6.7–6.9 (Sigma Aldrich 79383-250ML, Merck, Darmstadt, Germany) (1× PBS) and harvested by centrifugation at 14,000× *g* for 5 min. The cell pellet was either directly used for DNA extraction or stored at −20 °C. For DNA extraction, the PureLink Genomic DNA Mini Kit (Thermo Fisher Scientific Inc.) was used according to the manufacturer‘s instructions, using the Gram-negative bacteria genomic DNA purification protocol. Elution buffer EB (Qiagen 19086-250ML, Hilden, Germany) was used for DNA elution.

DNA concentration was quantified using a Qubit Fluorometer and the Qubit dsDNA BR Assay Kit (Thermo Fisher Scientific Inc.) according to the manufacturer’s instructions. DNA concentration was adjusted for real-time PCR analysis to 10 pg/µL with sonicated salmon sperm DNA (10 ng/µL) (Agilent Technologies, Santa Clara, CA, USA) used as background DNA.

### 2.4. Next-Generation Sequencing (NGS) and Assembly

For short-read sequencing, DNA libraries with an average insert size of about 400 bp were generated using the NEB (New England Biolabs GmbH, Frankfurt am Main, Germany) Ultra II DNA Library Prep Kit according to the manufacturer’s instructions and sequenced on the Illumina MiSeq benchtop sequencer using the MiSeq reagent kit v2 (2 × 150 bp, Illumina, Inc., San Diego, CA, USA). Paired-end reads were processed using the AQUAMIS pipeline v1.3.7 [35], which comprised quality control, trimming, and de novo assembly using Shovill. All assemblies fulfilled the quality criteria of Q30 for at least 75% and minimum coverage of 30×. The 21 Vietnamese isolates were sequenced and assembled at BfR as described in [20].

### 2.5. Design of Primers and Probes

Hundred *Campylobacter* isolates available at LGL (HS_1 to FS_100) and a worldwide collection of *Campylobacter* isolates from NCBI were used to design the oligonucleotides. Primers and probes were designed with the help of the NCBI Primer Blast Tool. An additional 29 German isolates from service laboratories in southern Germany and BfR (HS_101 to FS_129), as well as 21 Vietnamese isolates (VE_01 to VE_21), were applied for validation of the designed oligonucleotides.

Prevalent resistance determinants in *Campylobacter* isolates were retained to develop a pentaplex real-time PCR (multiplex real-time PCR with detection systems in 5 channels), allowing simultaneous detection of resistance genes and point mutations associated with tetracycline, ciprofloxacin, and erythromycin resistance. The IPC-ntb2 gene fragment from *Nicotiana tabacum* was used as internal amplification control (IAC, [36]) and extracted from *E. coli* DSM 116329 (DSMZ-German Collection of Microorganisms and Cell Cultures GmbH, Braunschweig, Germany). It was applied to detect PCR inhibition and to confirm negative results. Simultaneously, a triplex real-time PCR assay (3 fluorescence channels for *tet*(O), GyrA_T86I/V, and IAC) combined with a duplex real-time PCR assay (2 fluorescence channels for *erm(*B) and *23S rRNA_*A2075G) were validated in case of limited optical modules available in the real-time PCR instruments in user laboratories.

### 2.6. In Silico Screening for Primer Binding Sites and Gene Alignments

In silico primer screening [37] for the selection of designed primers and probes was performed to evaluate the specificity of the real-time PCR assay.

Assembly sequences were screened for primer and probe sequences using fastaRegexFinder [38].

NCBI reference sequences of resistance determinants (*gyrA C. coli*: GeneID: 66544015 *gyrA C. jejuni*: GeneID: 905319, *tet*(O) *C.jejuni*: GeneID: M18896.2) were blasted against a custom BlastDB based on all assembly sequences to identify and extract corresponding sequences from the assemblies. These were aligned using muscle 5.1 [39] and visualized with Aliview 1.2.6. [40].

### 2.7. Multiplex Real Time PCR Assay for Detection of Resistance Determinants

The real-time PCR assays were validated with QuantiNova Multiplex PCR master mix (Qiagen, Hilden, Germany) for the pentaplex assay in two different probe dye combinations, either FAM-ROX-Cy5-HEX-ATTO425 on AriaMx instrument (Agilent Technologies) or FAM-ROX-Cy5-HEX-Cy5.5 on Quantstudio5 (Thermo Fisher Scientific) and on CFX96 Touch System (Bio-Rad, Hercules, CA, USA). QuantiNova Multiplex PCR Kit was also appropriate for the combination of the triplex and duplex assays.

A total of 50 copies of the IPC-ntb2 plasmid [36] were added as IAC in the pentaplex and triplex assays. The reaction mix was filled with PCR-grade water to 20 µL. A volume of 5 µL DNA with a concentration of 10 pg/µL was added to the reaction mix. The protocols for all three reaction mix variations are given in Supplementary Material Tables S3–S6. Two Vietnamese isolates *C. coli* BfR-CA-15062 (VE_01, *tet*(O/M/O), GyrA_T86I, *erm*(B)) and *C. jejuni* BfR-CA-16092 (VE_14, *tet*(O/M/O) *+ tet*(O)*x*, GyrA_T86I, *23S rRNA*_A2075G) were used as positive control strains for the real-time PCR-assays.

The primer and the probe concentrations were optimized on the AriaMx instrument to achieve an optimal fluorescence signal for all primer–probe detection systems. The optimal annealing temperature of 60 °C was determined via a gradient PCR experiment on Quantstudio5 in which an annealing temperature gradient between 58 °C and 62 °C was applied. No significant differences were detected in real-time PCR results between 58 °C and 62 °C, but the fluorescence of the amplification curves was optimal for all detection systems at an annealing temperature of 60 °C. Amplification conditions with QuantiNova Multiplex PCR Kit on all three PCR instruments consisted of enzyme activation at 95 °C for 2 min followed by 40 cycles of 95 °C for 10 s, 60 °C for 20 s, and 72 °C for 20 s.

For the detection systems *tet*(O), *erm*(B), and IAC, labeled double-quenched probes were used, as they reduce background signals and crosstalk between the different channels of the real-time PCR instruments in multiplex PCR.

### 2.8. In-House Validation of the Pentaplex Real-Time PCR Assay

#### 2.8.1. Selectivity

The applicability of the pentaplex real-time PCR assay for detecting the resistance determinants was checked on all 129 DNAs of German *Campylobacter* isolates (HS_101 to FS_129) as well as on all 21 DNAs of Vietnamese isolates (VE_01 to VE_21) supplied by BfR.

#### 2.8.2. Determination of Efficiency and LOD_95%_

To access the efficiency and the limit of detection (LOD_95%_) of the detection systems for resistance determinants on AriaMx equipment (Agilent Technologies, Santa Clara, CA, USA), serial dilution of two *Campylobacter* isolates were applied to cover both erythromycin resistance determinants (*erm*(B) gene and the point mutation *23S rRNA*_A2075G), as well as both GyrA_T86I detection systems (*C. jejuni* and *C. coli*). *C. coli* isolate VE_01 with *erm*(B) gene and *C. jejuni* isolate VE_14 with *23S rRNA*_A2075G were selected.

The DNA copy number was adjusted to 5000 copies/µL DNA in ddPCR [41] based on an absolute quantification of DNA copy number. All DNAs were diluted to five dilution levels (5000, 1000, 500, 100, and 50 copies/µL DNA). Each dilution level was measured in three technical replicates to evaluate the efficiency of the pentaplex real-time PCR. The percentage of efficiency and the coefficient of determination R^2^ were calculated.

To determine the lowest copy number still detectable with a 95% confidence interval (LOD_95%_) a serial dilution of the target DNAs was prepared at 8 low copy number levels (20, 10, 4, 2, 1, 0.4, 0.2, and 0.02 copies/µL) and each dilution level was measured in 12 independent technical replicates. The probability of detection (POD curve) and LOD_95%_ was computed via a web service provided by QuoData (QuoData Web Service [42]) according to BVL guidelines [43,44].

#### 2.8.3. Robustness

The robustness of the real-time PCR assay was tested on two different real-time PCR machines from two additional manufacturers (Quantstudio5, Thermo Fisher Scientific, and CFX96 Touch System, Bio-Rad). The HiDi^®^ Taq DNA Polymerase and 10× buffer (MyPols Biotec, Konstanz, Germany) were used to check the suitability of a single components master mix in the real-time PCR assay. A 25 µL PCR reaction mix contained 1 × HiDi^®^ buffer, 2 IU per reaction of HiDi^®^ Taq DNA Polymerase, 1.5 mM MgCl_2_ (Thermo Fisher), 200 µM of each deoxynucleoside triphosphate (Takara Bio Inc., Kusatsu, Japan) and 5 mL of the sample DNA. The amplification conditions with HiDi^®^ Taq Polymerase were enzyme activation at 95 °C for 2 min followed by 40 cycles of 95 °C for 15 s, 60 °C for 30 s, and 72 °C for 30 s.

The combination of both factors (master mix/PCR equipment) was tested to detect potential effects on the real-time PCR performance. For this purpose, the efficiency was calculated using standard curves, as described in 2.8.2, with two *Campylobacter* isolates at five dilution levels (5000, 1000, 500, 100, and 50 copies/µL DNA).

## 3. Results

For the development of a multiplex real-time PCR assay, a test panel consisting of 129 *Campylobacter* isolates from Germany and 21 isolates from Vietnam was genotypically and phenotypically characterized for antimicrobial resistance. The correct assignment of phenotypic results (see Section 3.1) to genotypic results was verified in silico (see Section 3.3) and validated in the real-time PCR assay (see Section 3.4 and Section 3.5).

### 3.1. Antimicrobial Resistance Profiles

All 129 *Campylobacter* isolates from Germany were categorized into sensitive and resistant strains using the epidemiological cut-off values, which were based on the European Committee on Antimicrobial Susceptibility Testing and the European Food Safety Authority ([32,33,34], Table 1). The results of resistance profiles for all 129 isolates from Germany upon susceptibility testing against the six antimicrobials of the European-wide harmonized EUCAMP3 plate format are outlined in Supplementary Material Table S1 and summarized in Table 2. The two strains *C. jejuni* strain DSM 4688 and *C. coli* strain 2012-70-443-2 served as complete sensitive controls.

Resistance to gentamicin and chloramphenicol was not observed in German isolates. Food and human isolates were both predominantly resistant to ciprofloxacin (62.3 to 80.9%), followed by tetracycline (47.5 to 64.7%) and finally to ertapenem (17.6 to 18.0%). Resistance to erythromycin was observed always in combination with resistance to ciprofloxacin at a low level (1.6 to 2.9%) and only in *C. coli* isolates.

**Figure 1:**
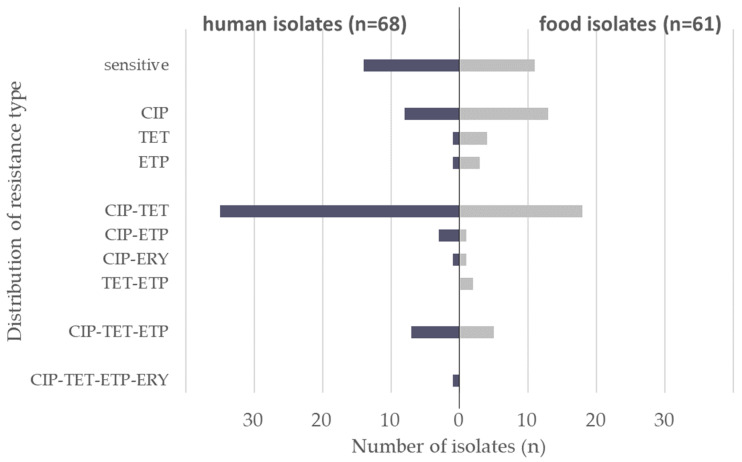
Distribution of resistance type (1-fold to 4-fold in EUCAMP3) for human and food isolates from Germany. HS, human isolates; FS, food isolates; CIP, ciprofloxacin; TET, tetracycline; ETP, ertapenem; ERY, erythromycin.

The distribution of combined resistance (1-fold to 4-fold) is displayed in Figure 1. In total, 23% of the German isolates showed resistance to a single antibiotic (*n* = 10 for humans, *n* = 20 for food). Overall, 47% of the isolates were resistant to two antibiotics in different combinations (*n* = 39 for humans, *n* = 22 for food). Finally, 9% of the isolates showed resistance to three antimicrobial agents (*n* = 7 for humans, *n* = 5 for food) and one human isolate showed resistance to the four antimicrobial agents ciprofloxacin, tetracycline, ertapenem, and erythromycin. The occurrence of combined resistance to ciprofloxacin and tetracycline is very frequent. Among 129 German isolates, 11 human isolates as well as 14 food isolates displayed no resistance to any of the six antibiotics tested in the EUCAMP3 panel.

### 3.2. Design of Primer and Probes for Real-Time PCR Assays

The pentaplex real-time PCR assay included four detection systems combined with the IAC. Four resistance determinants were detected simultaneously: suitable fragments of the resistance genes *tet*(O) and *erm*(B) as well as the point mutations GyrA_T86I/V and A2075G in the 23S rRNA gene.

For the point mutation GyrA_T86I, ATT in *C. coli* and ATA in *C. jejuni* codes for isoleucine, whereas in wild-type ACT in *C. coli* and ACA in *C. jejuni* codes the threonine. In point mutant A2075G in the 23S rRNA gene, contrary to the wild type, the base A is substituted with G. In all cases, labeled single-quenched probes with 4 LNA (Locked Nucleic Acid) bases [45,46,47] were used to stabilize hybridization and increase thermal stability. Additionally, unlabeled LNA probes with the wild-type nucleotide sequence were added, in order to improve specificity of the detection of *gyrA* and 23S rRNA gene-resistant mutants and to suppress the unspecific binding of the labeled LNA probes to wild-type sequences. The base sequences in *gyrA* for the point mutation T86I showed considerable differences between *C. coli* and *C. jejuni*; therefore, two different primer–probe sets were needed to screen ciprofloxacin resistance in both species simultaneously. Sequences and final concentrations of primers and probes (IDT, Coralville, IA, USA and metabion, Planegg, Germany) for pentaplex real-time PCR are listed in Table 3.

**Figure 2:**
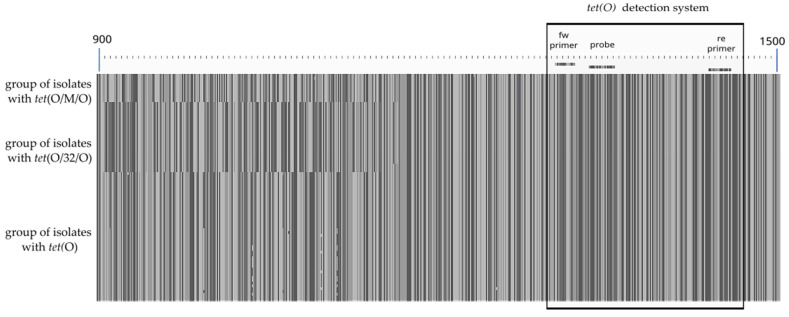
Detection system for *tet*(O) and *tet*(O) mosaic variants (Aliview 1.2.6.). From the top, 3 groups of isolates, with mosaic variant *tet*(O/M/O), with mosaic variant *tet*(O/32/O) and with gene *tet*(O). Figure 2 shows a segment of the alignment from nucleotides 900 to 1500. The detection system for *tet*(O) covers the nucleotides between 1304 and 1460. The complete sequence alignment extends over 1920 nucleotides.

Tetracycline resistance can be established by the presence of *tet*(O) and/or mosaic variants *tet*(O/M/O), and *tet*(O/32/O) [18,19]. The designed detection system for *tet*(O) delimited an area, similarly for all gene variants (Figure 2). The alignment of the sequenced tetracycline resistant *Campylobacter* isolates in Figure 2 shows the binding sites to *tet*(O) primers and probe, independently of *tet*(O) variants.

### 3.3. In Silico Screening in Comparison to Phenotypic Results

Binding sites were screened in silico for the designed primers and probes for the test panel to assess their ability to detect resistance genes and point mutations. Scanning the generated assemblies revealed the presence of binding sites in 94 isolates to *tet*(O), 10 isolates to *erm*(B), 12 isolates to A2075G point mutation in the 23S rRNA gene and 114 isolates (47 *C. coli*, 67 *C. jejuni*) to the GyrA_T86I mutation. The results of the binding site screening for the designed primer sets correlated with the results of the phenotypic resistance screening in EUCAMP3 (Table 4). The presence of primer and probe binding sites are summarized in Supplementary Material Table S2. For the two sensitive control strains *C. jejuni* strain DSM 4688 and *C. coli* strain 2012-70-443-2, no primer binding to the four designed resistance detection systems was predicted. For some assemblies based on short-read sequence data, the in silico screening predicted more than one copy of the *tet*(O) gene. This could be confirmed only with long-read sequencing, as shown in [20]. Since thermotolerant *Campylobacter* spp. harbor three copies of the ribosomal RNA operon, *23S rRNA* A2075G was occasionally detected as multiple copies in some of the assemblies [48].

**Figure 3:**
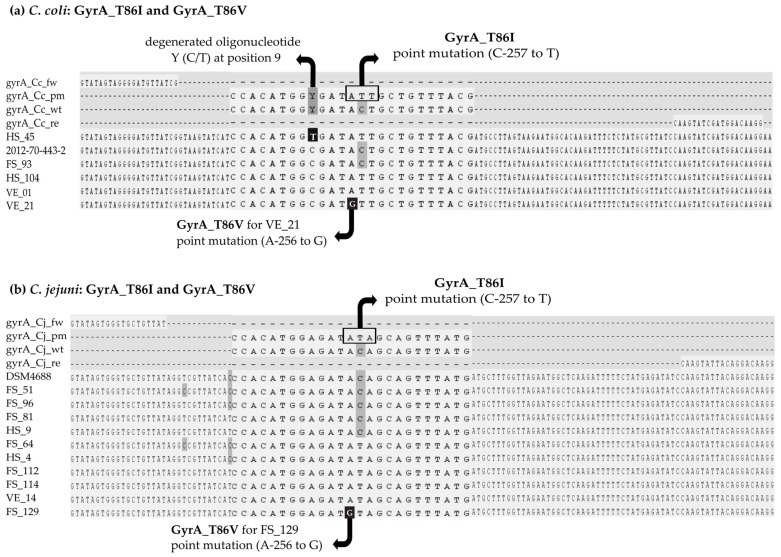
Primer binding sites for GyrA_T86I and GyrA_T86V of *C. coli* and *C. jejuni* (Aliview 1.2.6.); HS, human isolates; FS, food isolates; VE, Vietnamese food isolates; Cj, *C. jejuni*; Cc, *C. coli*.

The alignment of *Campylobacter* isolates in Figure 3 shows the binding sites to GyrA_T86I primers and probe for *C. coli* (a) and *C. jejuni* (b). A degenerated base Y (mixture of C and T) was integrated at position 9 of the probes for *gyrA_T86I*_*Cc*, to account for approximately 6% of *C. coli* isolates available in the NCBI nucleotide database (accession on 10 August 2022, determined by using NCBI Primer Blast Tool) that contain the base T instead of base C. The in silico screening was performed with corresponding alternative bases C (pm1) and T (pm2). In our study, 18 *C. coli* isolates showed base C and one human isolate showed base T (“**” in Table 4).

Ciprofloxacin-resistant food isolates FS_129 (*C. jejuni*, Germany) and VE_21 (*C. coli*, Vietnam) harbored an alternative mutation compared to the common ciprofloxacin resistant isolates resulting in GyrA_T86V for valine instead of GyrA_T86I for isoleucine. *C. coli* VE_21 showed the base triplet GTT (valine) instead of ATT (isoleucine) in *gyrA*. Likewise, *C. jejuni* FS_129 showed the base triplet GTA (valine) instead of ATA (isoleucine). The designed LNA probes did not account for this additional mutation (A-256 to G) (“+1*” in Table 4).

### 3.4. Multiplex Real-Time PCR Assay

The pentaplex real-time PCR assay was developed to detect simultaneously four resistance determinants, including the suitable fragments of the resistance genes *tet*(O) and *erm*(B) as well as the point mutations GyrA_T86I and A2075G in the 23S rRNA gene. The sequences and final concentrations of primers and probes (IDT and metabion) are listed in Table 3 as well as in Supplementary Material Tables S3 and S4. The designed detection system for *tet*(O) detected all isolates with tetracycline resistance, independently of the *tet*(O) variants (see also Figure 2). For the detection of the resistance to ciprofloxacin in *C. coli* isolates, the designed probes (*gyrA_T86I_Cc*) included a degenerated base Y (mixture of C and T) at position 9. As predicted in the in silico screening, all *C. coli* isolates with ciprofloxacin resistance (18 isolates with base C and one human isolate HS_45 with base T) were detected (see also Table 4, “**”).

In addition to the pentaplex real-time PCR assay, a triplex real-time PCR assay combined with a duplex real-time PCR assay consisting of the same primer and probe sequences but labeled with different fluorophores for detection were tested to allow usage of the system in case of limited optical modules in real-time PCR instruments (Supplementary Material Tables S5 and S6). The triplex real-time PCR method included two detection systems—resistance gene *tet*(O) in FAM channel (ZEN^TM^: internal quencher, IABkFQ: Iowa Black^®^ FQ quencher) and point mutation GyrA_T86I in ROX channel (BHQ1: Black Hole Quencher)—combined with IAC in HEX channel (ZEN^TM^: internal quencher, IABkFQ: Iowa Black^®^ FQ quencher). The duplex real-time PCR method covered the two resistance determinants for erythromycin resistance: resistance gene *erm*(B) in FAM channel (ZEN^TM^: internal quencher, IABkFQ: Iowa Black^®^ FQ quencher) and the point mutation *23S rRNA*_A2075G in HEX channel (BHQ1: Black Hole Quencher).

For evaluation of the real-time PCR assays, the threshold was set at about 10% of the maximum fluorescence of the positive control *C. coli* BfR-CA-15062 (VE_01) and *C. jejuni* BfR-CA-16092 (VE_14) for the four detection systems for resistance determinants and at 10% of the maximum fluorescence of the NTC (No Template Control) for the IAC. The triplex and duplex real-time PCR assays showed exactly the same PCR results as the pentaplex assay.

### 3.5. In-House Validation of the Multiplex Real-Time PCR Assay

#### 3.5.1. Specificity and Selectivity

The performance of the pentaplex real-time PCR assay in detecting resistance determinants was tested on all 129 *Campylobacter* isolates from Germany and on 21 *Campylobacter* isolates from Vietnam. The target specificity and selectivity of the real-time PCR were assessed by studying the inclusivity and exclusivity for all four resistance determinants. Isolates, for which antimicrobial resistance was phenotypically determined in the EUCAMP3 panel and resistance determinants were predicted genotypically via sequence analysis (NGS), were also positive for these determinants in the real-time PCR assays, showing 100% inclusivity. The cycle of quantification (Cq-values) for positive signals detected via real-time PCR on AriaMx equipment is presented in Table 5. Furthermore, isolates with phenotypic susceptibility and absence of resistance-determinants, predicted via NGS, were also negative in the real-time PCR assays, showing 100% exclusivity. No false-positive or false-negative signals were detected.

**Figure 4:**
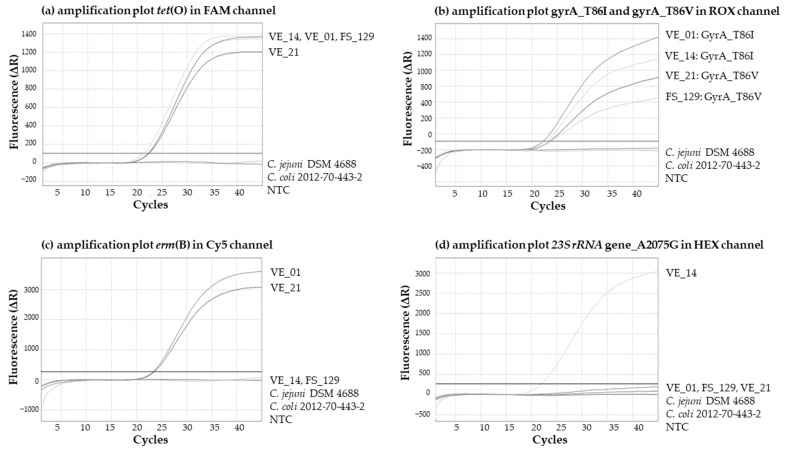
Amplification plots on AriaMx instrument for four resistant determinants. Real-time PCR detection of (**a**) *tet*(O); (**b**) both GyrA_T86I and GyrA_T86V mutation; (**c**) *erm*(B); (**d**) *23S rRNA*_A2075G mutation. Test strains harbored the following resistance determinants: BfR-CA-15062 (VE_01), *tet*(O)-*ermB*-GyrA_T86I; BfR-CA-16092 (VE_14), *tet*(O)-GyrA_T86I-*23S rRNA*_A2075G; FS_129, *tet*(O)-GyrA_T86V; VE_21, *tet*(O)-*ermB*-GyrA_T86V; DSM 4688 and 2012-70-443-2 served as negative controls for the four tested resistance determinants.

Amplification plots of the pentaplex real-time PCR-assay using AriaMx are presented in Figure 4. The two sensitive control strains *C. jejuni* strain DSM 4688 and *C. coli* strain 2012-70-443-2 were negative for the four detection systems for resistance determinants (Figure 4a–d). The positive control strains VE_01 and VE_14 were positive for the resistance determinants *tet*(O) and GyrA_T86I (Figure 4a,b). In addition, Figure 4c and Figure 4d highlight the difference in the erythromycin resistance determinant, *erm*(B) gene for VE_01, and the point mutation *23S rRNA*_A2075G for VE_14 respectively.

Two isolates FS_129 (*C. jejuni*, Germany) and VE_21 (*C. coli*, Vietnam) with the alternative mutation GyrA_T86V (see also Section 3.3 and “+1*” in Table 4) were detected in the ROX channel intended for the resistance determinant GyrA_T86I (Figure 4b). The base G (instead of A, Figure 3) did not interfere with the detection of the resistance-conferring mutation (GyrA_T86V).

#### 3.5.2. Determination of Efficiency and LOD_95%_

The efficiency of the pentaplex real-time PCR assay was investigated on five DNA concentrations (5000, 1000, 500, 100, and 50 copies/µL DNA) for two isolates. The linear regression analysis was performed, using AriaMx software Version 2.0. With a coefficient of determination R^2^ ≥ 0.98, the efficiency was 100% with less than ± 20% deviation from theoretical value. The designed primer–probe systems met the quality criteria of the BVL guidelines [43,44], as well as the Guidelines for validation of qualitative real-time PCR methods [49]. The results of efficiency tests are presented in Supplementary Material Table S7.

The LOD_95%_ for the four detection systems for resistance determinants was investigated by measuring 12 independent DNA replicates at eight low-copy-number levels (20, 10, 4, 2, 1, 0.4, 0.2, and 0.02 copies/µL) for two isolates. The LOD_95%_, the 95% confidence interval, and the mean probability of detection (POD) curve with respect to the corresponding 95% confidence range were computed via a web service provided by QuoData (QuoData Web Service [42). It was observed that the limit of detection for *tet*(O), GyrA_T86I for *C. coli* and *23S rRNA*_A2075G is slightly lower (LOD_95%_ ≤ 5 copies/µL) compared to *erm*(B) and GyrA_T86I for *C. jejuni* (LOD_95%_ ≤ 10 copies/µL) (Table 6).

#### 3.5.3. Robustness

The robustness of the pentaplex real-time PCR was evaluated by performing efficiency tests for the combination of two parameters, the real-time PCR equipment, and the master mix. Quantstudio5 and CFX96 Touch System as well as HiDi^®^ Taq DNA Polymerase master mix gave the same results as the ones obtained using the AriaMx real-time PCR equipment and QuantiNova Multiplex PCR master mix. The detection systems for the four resistance determinants met the quality criteria with an efficiency between 80 and 120% and a coefficient of determination of R^2^ ≥ 0.98 for all tested combinations. The real-time PCR assay was not influenced by the changes in the tested measurement conditions. The results of efficiency tests for the robustness are presented in Supplementary Material Table S7.

## 4. Discussion

*C. jejuni* and *C. coli* are the predominant *Campylobacter* species in poultry, causing a substantial impact on public health care and leading to most foodborne zoonotic diseases in humans. The prescription of antibiotics can be necessary to treat infections. Yet, the development of antimicrobial resistance (AMR) poses a steadily increasing problem by limiting the number of effective antibiotics. The European Centre for Disease Prevention and Control (ECDC) and the World Health Organization (WHO) have underlined the threat of antimicrobial resistance to patient safety and the need for global surveillance and concerted action throughout the European Region [50].

As van Belkum [51] presented in 2019, growth-based phenotypic analysis enables reliable antimicrobial susceptibility testing (AST) and ensures appropriate antibiotic therapy for infected patients. In our study, the EUCAMP3 microdilution panel was used for a reliable quantitative determination of the minimal inhibitory concentration (MIC) against relevant antimicrobials in food and human isolates of *C. jejuni* and *C. coli*. Among 129 German isolates, less than 20% were wholly susceptible to the six antibiotics tested. The most widespread antimicrobial resistance was against the fluoroquinolone ciprofloxacin. A high frequency of resistance to ciprofloxacin was also highlighted in the report of 2023 by the European Food Safety Authority (EFSA) and ECDC [29]. Combined resistance to ciprofloxacin and tetracycline was the most frequent resistance patterns observed in German human isolates and food isolates. In contrast, combined resistance to both ciprofloxacin and erythromycin, which is considered critically important for the treatment of campylobacteriosis [29], was not observed in *C. jejuni* and was rare in *C. coli* (8.3% for humans and 5.0% in food). The last two points were further reported in the report of EFSA and ECDC.

Comparing AMR against erythromycin, ciprofloxacin, and tetracycline in food and human isolates, no significant differences were detected in the frequency of antimicrobial resistance in our study. Moreover, due to the limited number of isolates no meaningful conclusion could be taken. This is in line with previous studies. McGill et al. [52] found similar resistance prevalence to erythromycin, ciprofloxacin, and tetracycline between food and human isolates in Ireland from 2001 to 2002. Similarly, in Estonia, Tedersoo et al. [53] found a comparable resistance to antibiotics for broiler chicken meat collected between 2018 and 2019 and human *Campylobacter* isolates. The appearance of resistant *Campylobacter* isolates in humans and animals likely reflects the wide use of antibiotics in poultry production. Yet, a few veterinary isolates (5 *C. jejuni*, 4 *C. coli*) (LGL) were investigated in EUCAMP3 and did not show a major divergence in the resistance profile compared to food and human isolates of this study. The prevalence of *Campylobacter* isolates with similar resistance profiles along the chicken food chain (high resistance rates to (fluoro-)quinolones and tetracycline and relatively low erythromycin resistance rate) was also shown in a German study from 2015 [54]. However, based on poultry data from 2014 to 2016 in Germany, Tenhagen et al. [55] demonstrated that antimicrobial resistance (AMR) and antimicrobial usage (AMU) cannot be systematically associated. Different factors, including animal species, two bacterial species (*C. jejuni* or *C. coli*), the antimicrobial agents, and the usage frequency (increase or decrease), should be further considered for a better understanding of the complex trends of the associations.

Since the early 2000s, genotypic-based methods, such as PCR assays, have been used to explore the determinants of antimicrobial resistance and are available as rapid screening methods to monitor and prevent the emergence of new bacterial antibiotic resistance. The qualitative pentaplex real-time PCR assay was developed based on the specific detection of four determinants in the current study. The elevated occurrence of resistance to ciprofloxacin and tetracycline in EUCAMP3 indicated the necessity to integrate two detection systems. First of all, the point mutation in *gyrA* led to the resistance-conferring amino acid exchange T86I in gyrase subunit A. Secondly, a detection system for the gene *tet*(O) included its *mosaic variants tet(O/M/O) and tet(O/32/O).* The frequency of these two resistance determinants was consistent with a previous study by Ghielmetti et al. [56], who illustrated an increasing prevalence of resistance to quinolones and tetracycline of *C. jejuni* isolates in Switzerland between 2003 and 2020. A combined resistance to both ciprofloxacin and erythromycin, which were considered crucial antimicrobials for the treatment of campylobacteriosis [50], was rarely detected in German isolates of this study (three isolates). Yet, it was frequently observed in isolates from Asian countries [57] and in 19 Vietnamese food isolates of the current study. To cover the resistance to erythromycin, a detection system for *erm*(B) and a detection system for the *A2075G s*ubstitution in the 23S rRNA gene were implemented in the real-time PCR assay. The gene *erm*(B) was exclusively detected in Vietnamese food isolates, whereas the A2075G point mutation in the 23S rRNA gene was detected in isolates from Germany and Vietnam. The pentaplex real-time PCR was successfully applied to DNA from all isolates of the test panel. The results of the complete test panel in the pentaplex real-time PCR correlated with the phenotypic results assessed in the EUCAMP3 panel and with genotypic results predicted by NGS data.

Due to the simultaneous detection of four resistance determinants in *C. jejuni* and *C. coli* within a single PCR reaction, the here-developed real-time PCR has an advantage over previously described singleplex conventional PCR systems [13,21,23,24]. Compared to previously described multiplex real-time PCRs [8,22], this PCR is adapted to the current prevalence of antibiotic resistance in human and food isolates from Germany. The pentaplex real-time PCR shows a limitation regarding the point mutations A2074C/G/T in the 23S rRNA gene*,* which is also associated with erythromycin resistance [12,13,23]. These point mutations could not be tested via the test panel. It can only be proven with appropriate isolates if the pentaplex real-time PCR detects these point mutations. If necessary, a new detection system should be integrated. Furthermore, a real-time PCR for ertapenem might be beneficial, as many German isolates show resistance against this antimicrobial agent (see Table 2). Yet, ertapenem is firstly not included in the priority panel for *Campylobacter* monitoring of human isolates at the EU level [29], and secondly, it exceeds the capability of the real-time PCR machine in the detection of more than five channels.

The developed multiplex PCR assay in this study improved the accuracy of analysis of antibiotics resistance in *Campylobacter*. However, challenges might exist, particularly when applied to the simultaneous detection of point mutations. All four detection systems were optimized for the same annealing temperature and showed similar PCR amplification efficiencies on different PCR machines. Therefore, the accurate detection of each target was not influenced by the other detection systems. These requirements ensured reproducible Cq-values between 21 and 26 on a fixed amount of DNA. Setting the threshold at around 10% of maximum fluorescence guaranteed comparable results for tested isolates. The developed pentaplex real-time PCR in this study, showed to be robust enough to be transferred to other real-time PCR machines combined with a different master mix. In addition, the developed method was a reliable, sensitive, and easily introducible screening method for the detection of AMR related to ciprofloxacin, tetracycline, and erythromycin resistance on isolates of *Campylobacter jejuni* and *coli*.

Our development can be implemented as a warning tool in routine analysis to detect the spreading of antibiotic resistance. A decisive advantage of real-time PCR assays is that the method can further be developed to detect new incoming resistance determinants. Finally, the real-time PCR assay as rapid qualitative screening tool in combination with EUCAMP as a phenotypic tool for quantifying resistance can be considered as excellent complementary methods.

## Figures and Tables

**Table 1:** Epidemiological cut-off values (ECOFFs) for evaluation of antibiotic susceptibility testing results of thermotolerant *Campylobacter* spp. from Germany.

Antimicrobial	MIC [mg/L] Resistant > *C. jejuni*	MIC [mg/L] Resistant > *C. coli*	Reference
ciprofloxacin	0.5	0.5	ECOFFs for *C.* spp. [32,33,34]
tetracycline	1	2	
ertapenem	0.5	0.5	
erythromycin	4	8	
chloramphenicol	16	16	
gentamicin	2	2	

MIC, minimum inhibitory concentration.

**Table 2:** Prevalence of phenotypic resistance of human and food isolates from Germany in EUCAMP3 panel.

	Percentage of German Isolates Resistant to Antimicrobials Tested (%)					
Antibiotic	Human Isolates (*n* = 68)			Food Isolates (*n* = 61)		
	*C. jejuni* (*n* = 44)	*C. coli* (*n* = 24)	*C. jejuni + C. coli*	*C. jejuni* (*n* = 41)	*C. coli* (*n* = 20)	*C. jejuni + C. coli*
ciprofloxacin	81.8	79.2	80.9	68.3	50.0	62.3
tetracycline	65.9	62.5	64.7	43.9	55.0	47.5
ertapenem	6.8	37.5	17.6	7.3	40.0	18.0
erythromycin	0.0	8.3	2.9	0.0	5.0	1.6
chloramphenicol	0.0	0.0	0.0	0.0	0.0	0.0
gentamicin	0.0	0.0	0.0	0.0	0.0	0.0

**Table 3:** Oligonucleotides for pentaplex assay: *tet*(O); GyrA_T86I; *erm*(B); *23S rRNA*_A2075G, IAC.

Antimicrobial Resistance and Target	Primer/Probe Name	Oligonucleotide Sequence 5’→ 3’	Amplicon Size [bp]	Final Concentration in qPCR [nM]	Reference
Tetracycline *tet*(O) ^1^	tet(O)-fw	AAGTCCCGCCAAATCCT	157 (Acc. No. NG_048257.1)	150 nM	the current study
	tet(O)-re	TGCTCGCAGCCATAAAGAA		150 nM	
	tet(O)-probe	**6-FAM **^6^— TCGGGTTGT*CCATAGAGCCG —IABkFQ ^12^		100 nM	
Ciprofloxacin for *C.jejuni* GyrA_T86I ^2^	gyrA_Cj_fw	GTATAGTGGGTGCTGTTAT	118 (Acc. No. wt AB104527.1, pm CP053659.1 )	400 nM	the current study
	gyrA_Cj_re	CCTTGTCCTGTAATACTTG		400 nM	[8]
	gyrA_Cj_wt	CCACATGGAGAT+A+**C**+A+GCAGTTTATG		600 nM	the current study
	gyrA_Cj_pm	**ROX **^7^—CCACATGGAGAT+A+**T**+A+GCAGTTTATG —BHQ2 ^13^		200 nM	
Ciprofloxacin for *C.coli* GyrA_T86I ^2^	gyrA_Cc_fw	GTATAGTAGGGGATGTTATCG	118 (Acc. No. wt CP092026.1 pm CP091310.1 CP082881.1)	400 nM	the current study
	gyrA_Cc_re	CCTTGTCCATCGATACTTG		400 nM	
	gyrA_Cc_wt	CCACATGGYGAT+A+**C**+T+GCTGTTTACG ^17^		600 nM	
	gyrA_Cc_pm	**ROX **^7^—CCACATGGYGAT+A+**T**+T+GCTGTTTACG —BHQ2 ^13, 17^		200 nM	
Erythromycin *erm*(B) ^3^	erm(B)-fw	AGGGTTGCTCTTGCACACTC	125 (Acc. No. MF134831.1)	400 nM	the current study
	erm(B)-re	GAACATCTGTGGTATGGCGG		400 nM	
	erm(B)-probe	**Cy5 **^8^—AGCTGCCAG*CGGAATGCTTTCA —IAbRQSp ^14^		200 nM	
Erythromycin *23S rRNA_* A2075G ^4^	23S_A2075G_fw	GTGGAGGTGAAAATTCCTC	113 (Acc. No. wt CP020776 pm GU384931.1)	400 nM	the current study
	23S_A2075G_re	CAAAGCCTCCCACCTATC		400 nM	
	23S_A2075G_wt	CAAGACGG+A+**A**+A+GACCCCGTG		600 nM	
	23S_A2075G_pm	**HEX **^9^— CAAGACGG+A+**G**+A+GACCCCGTG —BHQ1 ^15^		200 nM	
Internal PCR control (target gene ntb2 ^5^)	IPC-ntb2-fw	ACCACAATGCCAGAGTGACAAC	125	300 nM	[36]
	IPC-ntb2-re	TACCTGGTCTCCAGCTTTCAGTT		300 nM	
	IPC-ntb2 probe	**AriaMx: ATTO425 **^10^—CACGCGCAT*GAAGTTAGGGGACCA —IABkFQ ^12^ **QuantStudio5 and CFX96: Cy5.5 **^11^— CACGCGCAT*GAAGTTAGGGGACCA —NFQ-2 ^16^		150 nM	

^1^ Resistance gene *tet*(O); ^2^ point mutation in GyrA; ^3^ resistance gene *erm*(B); ^4^ point mutation in the 23S rRNA gene; ^5^ methyltransferase gene of *Nicotiana tabacum*; ^6^ FAM, 6-carboxyfluorescein; ^7^ ROX, carboxy-X-rhodamine; ^8^ Cy5, cyanine dye; ^9^ HEX, hexachlorofluorescein; ^10^ ATTO425, tetrazine dye**;** ^11^ Cy5.5, cyanine dye; ^12^ IABkFQ, Iowa Black^®^ FQ quencher; ^13^ BHQ2, Black Hole Quencher; ^14^ IAbRQSp, Iowa Black^®^ RQ quencher; ^15^ BHQ1, Black Hole Quencher; ^16^ NFQ-2, Non-Fluorescent quencher; ^17^ Y (C/T), degenerated nucleotide; +A, +G, +C, +T, base notation for Locked Nucleic Acid (LNA) bases; * = ZEN^TM^ or TAO or abNFQ-2 (internal quencher for FAM and ATTO425 or Cy5 or Cy5.5 respectively); point mutation in labeled probes (pm) and wild type in unlabeled probes (wt) are underlined.

**Table 4:** Correlation between phenotypic resistance results and primer binding sites (theoretical genotypic results).

		Tetracycline			Ciprofloxacin						Erythromycin	
			*tet*(O)		*gyrA_T86I*		*gyrA_T86I* *Cc*		*gyrA_T86I* *Cj*		*23S rRNA_* A2075G	*erm*(B)
		S	R	S	R	S	R	S	R	S	R	
DE	pheno	32	29	23	38	10	10	13	28	60	1	
(food)	geno	32	29	23	38	10	10	13	27 +1*	60	1	0
DE	pheno	24	44	13	55	5	19	8	36	66	2	
(human)	geno	24	44	13	55	5	19 **	8	36	66	2	0
VN	pheno	0	21	0	21	0	18	0	3	2	19	
(food)	geno	0	21	0	21	0	17 +1*	0	3	2	9	10

German (DE) and Vietnamese (VN) isolates from food and human origin; pheno, phenotypic result; geno, genotypic in silico result, S, sensitive; R, resistance; +1* no 100% concordance for two isolates with additional point mutation in *gyrA* (*C. coli* VE_21 and *C. jejuni* FS_129 with mutation GyrA_T86V for valine instead of GyrA_T86I for isoleucine); ** 18 *C. coli* isolates with binding site to probe *gyrA_T86I_Cc* pm1 (C) and one isolate with binding site to probe *gyrA_T86I_Cc* pm2 (T).

**Table 5:** Summary of pentaplex real-time PCR results.

Antimicrobial Resistance	Resistance Determinant/Gene	Channel	Prevalence of Positive Signals in PCR	Mean Cq-Value ± Standard Deviation	Range of Cq-Values
tetracycline	*tet*(O)	FAM	*n* = 94	23.36 ± 1.23	21.06–26.45
ciprofloxacin *C. coli*	GyrA_T86I *Cc*	ROX	*n* = 47	23.87 ± 1.26	22.12–26.92
ciprofloxacin *C. jejuni*	GyrA_T86I *Cj*	ROX	*n* = 67	24.05 ± 0.97	21.94–26.14
erythromycin	*23S rRNA*_A2075G	HEX	*n* = 12	22.48 ± 0.75	21.54–23.92
erythromycin	*erm*(B)	Cy5	*n* = 10	24.10 ± 1.11	23.24–26.62
IAC	ntb2	ATTO425	*n* = 150	31.56 ± 0.46	30.67–33.10

Cq, cycle of quantification.

**Table 6:** Results of LOD_95%_.

	BfR-CA-16092 (VE_14, *C. jejuni)*		BfR-CA-15062 (VE_01, *C. coli)*	
	LOD_95%_	95% Confidence Interval	LOD_95%_	95% Confidence Interval
*tet*(O)	1.460 cp/µL	[0.961, 2.219]	2.533 cp/µL	[2.028, 5.229]
*gyrA*_T86I *C. jejuni*	6.115 cp/µL	[4.134, 9.093]		
*gyrA*_T86I *C. coli*			1.696 cp/µL	[1.119, 2.565]
*23S rRNA*_A2075G	1.214 cp/µL	[0.928, 2.265]		
*erm*(B)			5.835 cp/µL	[3.938, 8.663]

## Data Availability

Data are contained within the article and supplementary materials.

## Supplementary Materials

The following supporting information can be downloaded at: https://www.mdpi.com/article/10.3390/microorganisms11122927/s1. Table S1: Sample overview, including phenotypic resistance determined with EUCAMP3 and resistance determinants relevant for real-time PCR assay. Table S2: Primer binding sites. Table S3: Protocols for pentaplex real-time PCR assay on AriaMx. Table S4: Protocols for pentaplex real-time PCR assay on CFX96 Touch System and Quantstudio5. Table S5: Protocols for triplex real-time PCR assay. Table S6: Protocols for duplex real-time PCR assay. Table S7: Results of efficiency and robustness tests for pentaplex real-time PCR assay.
